# Enrolling a rural community pharmacy as a Vaccines for Children provider to increase HPV vaccination: a feasibility study

**DOI:** 10.1186/s12889-021-11304-8

**Published:** 2021-06-29

**Authors:** Casey L. Daniel, Frances Lawson, Macy Vickers, Chelsea Green, Anna Wright, Tamera Coyne-Beasley, Hee Y. Lee, Stacie Turberville

**Affiliations:** 1 Division of Cancer Control and Prevention, Mitchell Cancer Institute, University of South Alabama, 1660 Springhill Avenue, Mobile, AL 36604 USA; 2 Division of Adolescent Medicine, University of Alabama at Birmingham, Birmingham, AL USA; 3 School of Social Work, University of Alabama, Tuscaloosa, AL USA

**Keywords:** HPV, Human papillomavirus, Vaccination, Cancer, Prevention, Pharmacy

## Abstract

**Background:**

Human papillomavirus (HPV) is the most common sexually transmitted infection in the U.S. with over 80 million infected individuals. High-risk strains are associated with 6 different cancers. Although infection is preventable, U.S. vaccination rates remain suboptimal and there are noted disparities between urban and rural communities due to economic barriers, lack of access, and low awareness and education.

**Methods:**

The current pilot study sought to overcome these barriers through an interprofessional collaborative enrolling a community pharmacy in a rural, medically underserved Alabama county as a Vaccines for Children (VFC) provider to provide free vaccines to eligible adolescents. Program evaluation was conducted to determine the intervention’s feasibility. Potential efficacy was assessed by analyzing county-level HPV vaccination uptake and completion rates using state immunization registry data.

**Results:**

Over the 8-month study, 166 total vaccines were administered to 89 adolescents ages 10–18, including 55 doses of HPV vaccine, 53 doses of Tdap vaccine, 45 doses of meningococcal vaccine, and 13 doses of influenza vaccine. Among these adolescents, mean age was 12.6 years old, and 64 (71.9%) were VFC patients. The pharmacy recorded an increase in total vaccine administration of 158.8%, an increase in prescription revenue of 34.8%, and an increase in total revenue by 24.4% during the course of the study, compared to the previous year.

**Conclusions:**

Findings from the current work demonstrate the potential of this strategy and can serve as a blueprint for statewide and national dissemination and implementation to ultimately increase access to vaccination services, increase vaccination rates, and reduce urban-rural vaccine disparities.

## Contributions to the literature

- The current study suggests a new and innovative way to administer the HPV vaccine to medically underserved populations utilizing rural, community pharmacies, which to our knowledge has not been previously investigated in the literature.
- This study uses a novel approach of administering HPV vaccines through pharmacies registered in the federally funded VFC program. We propose that this method will increase accessibility of the HPV vaccine, as it provides free vaccines for Medicaid or Medicaid-eligible children.
- These findings demonstrate the potential of this strategy and suggest that it would be successful in increasing HPV vaccination rates on a broader scale.

## Background

HPV vaccination is extremely effective at preventing infection with high-risk, cancer-causing strains of HPV [1]. The HPV vaccine, administered in a 2- or 3-dose series (depending on age of first dose) is recommended for males and females between the ages of 9–26 years old and has recently been approved by the FDA for adults up to age 45 [2, 3]. Since its introduction in 2006, rates of HPV vaccination in the U.S. have improved, but remain far below the Healthy People 2020 goal of 80% coverage for adolescents age 13–15 [3]. HPV vaccination rates in the U.S. also lag behind comparably industrialized countries like Australia which, in 2017, reached a completion rate of 75.9% in males and 80.2% in females by 15 years of age [4]. Low education, low awareness of HPV vaccination, lack of strong provider recommendations and barriers to appropriate healthcare are cited as some of the most prevalent reasons for low uptake and completion in the U.S. [4–6]. Rural settings are particularly vulnerable to these barriers with studies demonstrating that rural populations are less likely to be vaccinated [7, 8]. Rural and urban disparities in HPV vaccination have emerged as a concern, with approximately 60 million Americans (almost one-fifth of the population) living in rural locations [9, 10]. Recent research efforts have sought to determine ways to reduce these barriers and improve HPV vaccination specifically in rural populations [7, 11–13].

Among the most promising opportunities to increase HPV vaccination rates, particularly in rural settings, is by utilizing pharmacies as alternative vaccination sites [7, 12]. There is a wealth of existing literature advocating for pharmacies to serve in this capacity to reduce barriers and improve access to HPV vaccination, including the 2018 President’s Cancer Panel report on HPV vaccination [14–16]. Some existing resources unique to many pharmacies making them ideal alternative settings for vaccination include: infrastructure and storage capacity for various medications [17], established vaccine protocols [17, 18], longer hours than traditional healthcare provider offices as well as weekend availability [19–21], and convenience/greater accessibility [12, 18, 22]. In 2016, the U.S. National Vaccine Program Office reported that 95% of Americans lived within five miles of a community pharmacy, removing a critical access barrier for rural populations that have to travel substantial distances to reach a provider [13]. Close provider proximity is additionally beneficial for vaccines such as the HPV vaccine which consists of multiple doses, increasing the likelihood that patients will follow-up to complete the series [23]. Community pharmacies (frequently independently owned and operated versus a large chain store) are particularly important for consideration as alternative settings for HPV vaccination. In rural and small-town settings, these pharmacies are frequently an established part of the community. Community pharmacists are often well-known, trusted members of the community who act as first-line healthcare providers [7].

Pilot studies have looked at implementation of interventions offering HPV vaccination in pharmacy settings. In 2018, Michigan researchers implemented a pharmacy-based pilot in 10 retail pharmacies, with the objective of identifying barriers, challenges, and successes with regard to HPV vaccination. Major findings included barriers specific to the HPV vaccine (specific age range, social stigma, the anti-vaccination movement) that were distinct from barriers to the influenza vaccine; low pharmacist confidence in recommending the vaccine; and high cost for patients. At roughly $250 per dose, cost is a significant barrier to overcome without assistance programs [24]. In the most recent study, performed by Calo et al. in 2019, HPV vaccination protocols were implemented in 15 clinics throughout five states. However, only 13 doses of the HPV vaccine were given over a span of 1 year. Researchers identified various explanations for the significantly underperforming results, including low parent demand, low engagement among pharmacy staff, poor-third party reimbursement, and limited integration into primary care systems [25].

To our knowledge, no studies have focused on rural community pharmacies. Rural populations are often medically underserved and overlooked in research [7]. Considering 20% of the American population lives in rural areas, it is imperative that efforts be made to address their significant barriers to healthcare [10, 11]. One barrier is lack of access to care; for example, 22 of 67 counties in Alabama, a largely rural state, lack a pediatrician and 2 counties do not have any primary care providers [26]. Although previous pharmacy studies have focused on increasing access to HPV vaccination, most have not addressed the frequently inextricable economic barriers that many in rural settings face [8]. Individuals in rural areas are more likely to be poor and more likely to rely on Medicaid than their urban counterparts [27, 28]. Children on Medicaid have limited vaccination options because they can only receive immunizations from Vaccines for Children (VFC) providers. The VFC program provides free vaccinations to children who might not otherwise receive the vaccine because of inability to pay [29]. Therefore, increasing access points alone in rural settings does not adequately address the needs of these populations. Additional vaccination settings must be approved VFC providers if they are to effectively serve the needs of their population [8]. The current pilot study examined the feasibility and efficacy potential of enrolling a rural, community pharmacy as a VFC provider to increase access and promotion of HPV vaccination.

## Methods

We identified an independent, community pharmacy partner within a county in Alabama meeting the study inclusion criteria [30]. The county selection criteria consisted of: designation of the county as noncore and rural according to the United States Department of Agriculture (USDA) standards [31], lacking a practicing pediatrician [32], 20 or fewer primary care providers [26], 12 or fewer VFC providers [33], number of pharmacies (emphasizing independent, community pharmacies), percentage of Medicaid-eligible children/adolescents greater than 50% [32], county demographics including having at least 2000 adolescents ages 10–18 years old [32], relative proximity to the study team (within 200 miles), and HPV vaccine initiation and completion rates below the state average [34]. A comprehensive list of all registered pharmacies in Alabama was provided by the Alabama Board of Pharmacy. Clarke County, Alabama was ultimately selected based on these criteria, and we established a collaboration with an independent, community pharmacy within the county. The target population for the current intervention were VFC-eligible/enrolled male and female adolescents age 11–15 living in Clarke County, Alabama. The primary outcome of the study was HPV vaccines administered with the secondary outcome of administration of other adolescent vaccines.

We next conducted formative research and a needs assessment, using a mixed methods research design, to develop a better understanding of the surrounding communities, local norms, existing resources, gaps, and needs. Additionally, we assessed the pharmacy, its customer base, infrastructure, capacity, staff, perceptions and attitudes. For this formative research phase, we utilized quantitative data collected through our initial county selection process as well as qualitative interviews conducted with members of the community and individuals at the pharmacy. Our most extensive qualitative interviews were conducted with the pharmacy owner/pharmacist.

At the time of this study, only one pharmacy in the state of Alabama was enrolled as a VFC provider and other pharmacies were not eligible for VFC enrollment [33]. However, we received approval for the study from the Immunization Division of the Alabama Department of Public Health (ADPH), including authorization to assist our pharmacy partner with enrolling as a VFC provider. We assisted the pharmacy in all aspects of the VFC enrollment process including: obtaining a physician standing order for HPV vaccination, scheduling training for Alabama’s state immunization registry (Immunization Division Patient Resources with Integrated Technology—ImmPRINT), completion of the extensive VFC provider application, coordination with ADPH, and—after receiving approval—ordering vaccines for VFC administration. We also worked with Alabama Medicaid to ensure that a pharmacy VFC provider would receive appropriate Medicaid reimbursement fees associated with VFC vaccination.

Another key element of the current study was the development and execution of a health communication campaign targeting parents of adolescents throughout the county to increase knowledge and awareness regarding HPV and HPV vaccination, as well as to promote HPV vaccination. The campaign, its messaging, points of emphasis, and channels of communication were informed by the data collected during the formative research phase and founded in constructs of the Health Belief Model [35]. It included digital and print materials consisting of similar facts and messaging, all developed by the research team. Key elements of this campaign included a large printed mailer sent to all households with an adolescent(s) aged 10–18 in residence, large posters on display locally, informational pamphlets, information cards that were stapled to prescription bags of relevant individuals, a social media strategy, and follow-up cards for adolescents who were vaccinated to remind them when to come back for their next dose. These materials were designed to be culturally relevant and targeted to individuals living in Clarke County by emphasizing facts and statistics specific to the population. Prior to start of the 2019 academic school year, we held a community back to school vaccine clinic and block party at the pharmacy. At this event, in addition to games and prizes, we distributed school supplies, educational information regarding HPV and HPV vaccination, and vaccines were offered.

Once VFC vaccine administration began at the collaborating pharmacy, we conducted ongoing process evaluation through site visits and regular calls with the pharmacy owner and pharmacist. The data collection period was originally planned for July 2019–August 2020. However, due to the unprecedented COVID-19 pandemic, the pharmacy had to halt vaccine administration for approximately 2 months due to state- and locally-mandated quarantine periods. Therefore, the study period was adjusted from July 2019–March 2020. Vaccinations resumed in June 2020. Evaluated items included: vaccine administration protocol (from consultation through data entry into ImmPRINT), distribution of in-house print materials and upkeep of social media, patron and community feedback, challenges encountered, solutions to these, issues related to claims and reimbursements, assistance needed, and other general feedback.

Vaccination rates were monitored throughout the study period through the ImmPRINT registry system. The primary outcome was number of HPV vaccines administered, though we also collected data on other adolescent vaccinations recommended by the CDC Advisory Committee on Immunization Practices (ACIP) that were administered at the pharmacy including tetanus, diphtheria, and pertussis (Tdap), meningococcal, and influenza [36]. The pharmacy ordered both VFC and non-VFC stock of these vaccines and they were maintained separately by the pharmacist. Additionally, we assessed the impacts of the intervention on the pharmacy from a business perspective including potential influences on foot traffic, new patients, overall revenue, prescription volume, public perception, and other outcomes to determine potentially unforeseen influences of the intervention.

## Results

Table 1 displays demographics for Clarke County, Alabama at the time of the study. The county was designated as noncore and rural by USDA, with a total population of 24,392 individuals, 5342 (21.9%) of whom were under the age of 18 years [37], and a population of 20.9 individuals per square mile [37, 38]. With respect to race, 52.8% were White, 44.0% Black/African American [38]. Among residents ages 25 and older, 80.6% had received a high school diploma or higher; 12.2% had a bachelor’s degree or higher [38]. Median household income for Clarke County was $34,100, with 39% of children in poverty [38], and 70% of children eligible for Medicaid annually [39]. Among Clarke County adolescents ages 13–17, 27% had initiated HPV vaccination (received at least one dose), with only 9% having completed the series, making this the lowest HPV vaccine completion rate of all 67 Alabama counties [34]. Additionally, Clarke County did not have a practicing pediatrician [32]. At the time of intervention, the only other location in the city for HPV vaccination was the county health department, which was experiencing difficulty meeting vaccination needs due to understaffing.

**Table 1:** Clarke County Demographics and Inclusion Criteria [31–34, 37–39]

Variable	N (%)
**Population**	
Total population	24,392
< 18 years	5114 (21.9%)
**Race/Ethnicity**	
White	12,879 (52.8%)
Black	10,733 (44.0%)
Hispanic/Latino	348 (1.4%)
**Median Household Income**	$34,100
**Annual Medicaid Eligible Children**	4200 (70%)
**Educational Attainment**	
High school graduate or equivalent	80.6%
Bachelor’s degree or higher	12.2%
**County Type**	Noncore
**HPV Vaccination Rates**	
Initiation	27%
Completion	9%
**Number of Providers**	
Primary Care Physicians	14
Pediatricians	0
VFC Providers	10
Independent Pharmacies	4
Chain Pharmacies	6

### Formative research

In addition to the quantitative data reported above, qualitative interviewing provided extensive data regarding the selected pharmacy, its resources and potential needs, and the owner/pharmacist (ST)’s experience and perspective. One finding was the confirmation that Grove Hill, the town in which the intervention pharmacy is located, consisted of a large Medicaid population and was in need of increased VFC services within the community. This was determined through discussion about the lack of healthcare resources available to residents. While the town had a few primary care physicians, none were practicing in the town full-time (rather, only 1–2 days per week) at the time of the study. No pediatricians were located within the county. As a result, residents of Grove Hill frequently had to travel to neighboring cities and towns to seek pediatric and adolescent healthcare serving Medicaid patients. At the start of the study, the only VFC provider located within the town was a health department location; however, this facility did not have any available vaccination appointments for the next several months.

Prior to the study, the intervention pharmacy had only administered adult vaccinations, and had never administered the HPV vaccination. The pharmacy’s vaccination capacity had been previously limited due to the aforementioned large Medicaid population in the community and not being a VFC-enrolled provider. ST emphasized many advantages of being able to provide VFC vaccinations at her pharmacy, including increasing accessibility and convenience. ST stressed her belief that patients would utilize these increased vaccination services, in part, due to the tightknit community that is characteristic of many small towns. ST also emphasized the value of the well-established trust and comfort level of her patients, promoting uptake. An additional advantage of enrolling this pharmacy as a VFC provider was its valued familiarity as a part of the local community, rather than providers who practiced locally but did not live within the town and/or were not engaged in the community. Additionally, ST indicated that community pharmacists in rural areas such as herself are trusted healthcare providers and frequently utilized as the first-line of healthcare for local residents who face barriers such as driving an hour or more to the closest pediatrician, or concern about expending one of their limited Medicaid office visits. Qualitative data from these interviews were consistent with the quantitative data previously collected, confirming that additional options were greatly needed for residents of this high-poverty county, particularly the critical health service of child and adolescent vaccinations.

### Process evaluation

As previously stated, the data collection period for the study was originally planned for July 2019–August 2020 but was adjusted to encompass July 2019–March 2020 due to the unprecedented COVID-19 pandemic, which resulted in halting vaccine administration for approximately 2 months. Vaccinations resumed in June 2020. Evaluated items included: vaccine administration protocol (from consultation through data entry into ImmPRINT), distribution of in-house print materials and upkeep of social media, patron and community feedback, challenges encountered, solutions to these issues related to claims and reimbursements, assistance needed, and other general feedback. At the time of the study, Alabama’s VFC enrollment process had many barriers for interested providers and was not designed or equipped for healthcare providers outside of traditional physician settings, making this pharmacy’s enrollment laborious and extremely time-consuming. Because of invalid links and lack of clear program expectations for providers, the pharmacist expressed significant concern about inadvertently erring in the enrollment and vaccine management processes which could result in fines up to $50,000. Fear of an accidental misstep was a significant deterrent to pursuing enrollment and required many communications with ADPH for clarifications. We also served as a liaison between VFC representatives and the pharmacy to facilitate enrollment, assist with coordinating site visits, and resolve issues and inquiries.

After successfully enrolling the current pharmacy as a VFC provider, the study team provided an extensive summary of suggested revisions to ADPH with respect to its website. These included a simplified website layout explicitly stating the steps to become a VFC provider, concise information about the yearly requirements to comply with CDC regulations, and detailed checklists for each. Additionally, links for vaccination refrigerator temperature logs, replacement policies, logging instructions, and storage care were streamlined and presented in a simplified manner to increase accessibility and feasibility for interested and participating providers. ADPH adopted the majority of these suggested revisions, improving navigation and clarity for individuals seeking VFC enrollment.

### Outcome evaluation

Table 2 displays the number of HPV, tetanus, diphtheria, and pertussis (Tdap), meningococcal, and influenza vaccinations administered to individuals ages 10–18 from July 29, 2019 to March 10, 2020. In this time period, 166 vaccines were administered to 89 adolescents. Of these, 51 (57.3%) were female, 56 (62.9%) were Black/African American (33.7% White), and mean age was 12.6 years old; 64 (71.9%) were VFC patients (Table 3). Among these adolescents, 84 (94.4%) were eligible for HPV vaccination, having not yet initiated and/or completed the series. Of the 166 total vaccines administered, 55 (33.1%) were HPV, 53 (31.9%) were Tdap, 45 (27.1%) were meningococcal, and 13 (7.8%) were influenza. The 55 HPV vaccines were administered to 54 adolescents; 46 (83.6%) of the HPV vaccines administered were initial doses. In this 8-month period, only one adolescent received both initiation and completion doses of HPV vaccine (many were not eligible to receive both due to the required time lapse between doses – completion occurring after the time lapse was also undetermined due to limitations by COVID-19). ST monitored initiation or completion doses was through patient interviewing and immunization registry confirmation prior to vaccine administration. ST provided reminder cards (designed for the current study) to patients requiring a completion dose listing the date of the initial vaccination and the date they should return for their completion dose.

**Table 2:** Preliminary Vaccination Data: July 29, 2019-March 10, 2020 for children and adolescents ages 10-18

	Private Insurance N (%)	VFC N (%)	Total
**HPV**	9 (16.4)	46 (83.6)	55
**Meningitis**	14 (31.1)	31 (68.9)	45
**Tdap**	13 (24.5)	40 (75.5)	53
**IIV4/IIV4-P Free**	7 (53.8)	6 (46.2)	13

**Table 3:** Demographic characteristics of adolescents who received vaccinations at the community pharmacy

Variable	N (%)
**Sex**	
Male	38 (42.7)
Female	51 (57.3)
**Age at Vax**	
10 years old	2 (2.3)
11 years old	44 (49.4)
12 years old	19 (21.4)
13 years old	5 (5.6)
14 years old	3 (3.4)
15 years old	0 (0.0)
16 years old	3 (3.4)
17 years old	4 (4.5)
18 years old	9 (10.1)
**Race**	
Black/African American	56 (62.9)
White	30 (33.7)
Other	3 (3.4)
**VFC patient**	
No	25 (28.1)
Yes	64 (71.9)
**Received Tdap**^**a**^	53 (60.0)
**Received MCV40**^**a**^	45 (50.6)
**Received Flu**^**a**^	13 (14.6)
**Received Any Dose HPV**^**a**^	54 (60.7)
**Received HPV Dose 1**	46 (51.7)
**Received HPV Completion Dose**	9 (10.1)

^a^Receipt of these vaccinations are not mutually exclusive

**Fig. 1:**
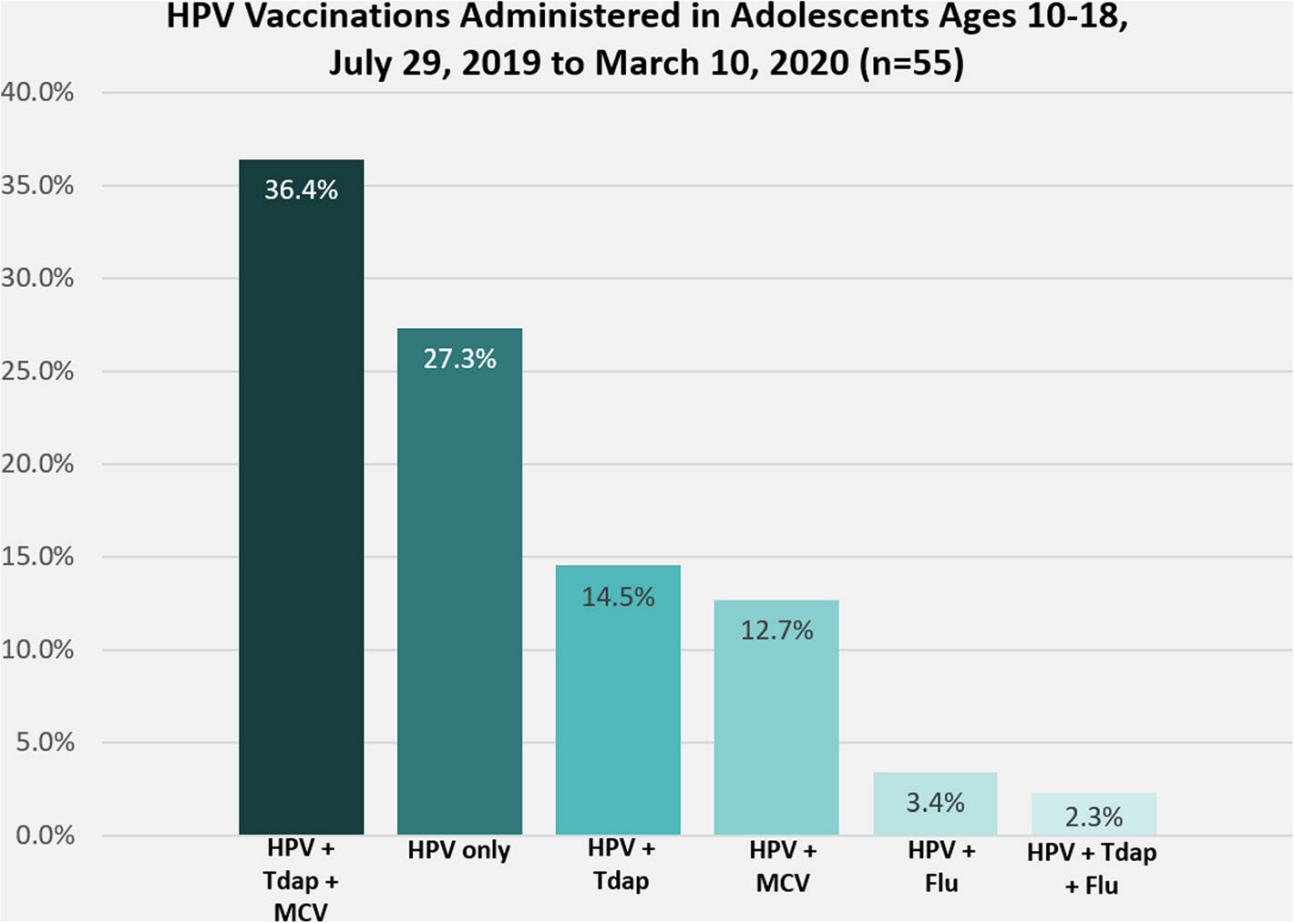
HPV vaccinations administered to adolescents ages 10-18 from July 29, 2019-March 10, 2020 (*n*=55)

Of the 54 adolescents who received HPV vaccination at the pharmacy, 33 (61.1%) were female, 33 (61.1%) were Black/African American (35.2% White), and mean age was 12.4 years old; 45 (83.3%) were VFC. Fifteen (27.3%) HPV vaccines were administered without other vaccines, 20 (36.4%) were given concomitantly with both Tdap and meningococcal vaccines, 8 (14.5%) were administered with Tdap alone, 7 (12.7%) were given with meningococcal alone, 3 (3.4%) were administered in conjunction with a flu vaccine; 2 (2.3%) were given with some other combination of these vaccines (Fig. 1). According to data from ADPH, HPV vaccination coverage in this county increased from 27% in 2018 to 34.4% in 2019 among adolescents ages 11–13 years.

From July 2019 to June 2020, the pharmacy administered a total of 440 vaccines—a 158.8% increase in vaccinations from the prior year compared with a 22.3% vaccination increase from the previous year. Also of note, the pharmacy experienced significant growth in additional services. In the 5 months following the start of the intervention, the pharmacy increased overall prescription revenue by 34.1% (compared to a 6.9% increase for this time period in the previous year). From July 2019 to June 2020, the pharmacy demonstrated a 17.8% increase in Medicaid prescriptions filled, thought to be heavily influenced by the added Medicaid/VFC services. Additionally, total revenue increased 24.4% after introduction of the intervention (August 2019 to March 2020), compared to an 8.0% increase the previous year. The pharmacy also noted significant increases in foot traffic.

Additionally, the pharmacy experienced shifts on the interpersonal and community levels, receiving positive feedback and recognition from the community as a whole. ST noted significant increases in outward, positive public perception particularly after hosting a vaccine clinic paired with a, “Back to School Block Party.” Following this event, ST received many, significant expressions of appreciation from both members and organizations within the community, emphasizing gratitude for the pharmacy’s community engagement and services. The intervention was also received positively by other local healthcare providers, with ST noting receiving vaccination referrals from some providers in the area as well as the health department.

Another outcome of the study was reaching an agreement with Medicaid of Alabama to provide administrative reimbursement costs to the pharmacy for VFC vaccinations. These costs were initiated in November 2019; however, the study team assisted in negotiating for retroactive reimbursements for VFC vaccinations administered from the start of the program.

## Discussion

The purpose of the current study was to examine the feasibility and potential effectiveness of enrolling a rural, community pharmacy as a VFC provider to increase access to HPV vaccination in a rural, medically underserved Alabama community. Our findings indicate strong potential for the feasibility and success of this intervention, as well as capacity for its broader dissemination and implementation to increase HPV vaccine knowledge, awareness, and rates of vaccination, particularly within rural populations.

Despite the study period being halted due to the COVID-19 pandemic and subsequent quarantines and temporary shut downs, the pharmacy administered 55 HPV vaccines to 54 adolescents ages 10–18 in under 8 months. This is a substantial achievement, particularly given the population of the city of Grove Hill, which was estimated to be 1573 in 2018 [40]. The success achieved here is outstanding compared to previous pharmacy interventions. In a pilot project involving 15 pharmacies across five states by Calo et al., only 13 HPV vaccine doses were administered to adolescents over the combined 12-month study period [25]. The authors identified the primary reasons for these poor outcomes as low parent demand and engagement among pharmacy staff as well as administrative barriers, including lacking third-party reimbursement [25]. Researchers in Michigan conducted a similar pilot study in ten pharmacies, and pharmacists administered only three doses of the HPV vaccine over a three-month period. Major barriers identified by this study included low pharmacist confidence in recommending the vaccine and high cost [41]. It is likely that the current study was significantly more successful because of the targeted approach, high engagement of the pharmacy staff, and—perhaps most importantly—because of the VFC provider enrollment facet which assisted with costs and availability. Additionally, our work securing Alabama Medicaid’s payment administrative reimbursement fees for the VFC vaccines administered by the pharmacy was a key achievement and contributor to the sustainability of the program.

The intervention addressed a number of commonly cited barriers to HPV vaccination, most significantly access to care, cost, convenience, and receiving a strong vaccine recommendation [7, 42, 43]. Offering the vaccine in the venue of a community pharmacy frequented by many local residents substantially improved access to care for this population that lacked dedicated pediatric/adolescent healthcare providers within the county. As anticipated, patients’ familiarity and comfort with the pharmacy and pharmacists also contributed to the success of the intervention. Many of the pharmacy’s long-time patients stated their inclination for HPV vaccination because of established trust in ST and her recommendations.

This intervention also overcame some of the socioeconomic and convenience obstacles associated with HPV vaccination. Having the vaccine so accessible removed barriers such as having transportation to reach a pediatric/adolescent healthcare provider (several miles away at minimum). Also, with hours extending beyond those of a typical provider office, the pharmacy offered broader windows for vaccination, presenting an opportunity for parents to take their children for vaccination without having to take time off of work (a particular barrier for many low-income families) for travel and appointments for what they may perceive as a nonessential vaccine. This also took into account the many adolescents who only present at a provider’s office for *required* vaccines, sports physicals, and/or when sick, as adolescents have demonstrated inconsistency in well-visit attendance [44]. The pharmacy provided additional convenience in that a pre-scheduled appointment was not necessary for vaccination, administration was quick, and the location was easy to get in and out of for patients.

The element of VFC enrollment was critical to the success of the intervention by addressing one of the most fundamental needs of this specific population, demonstrated by the fact that over 80% of those who received HPV vaccination were VFC patients. VFC-enrolled children and adolescents can only receive vaccines from a designated VFC provider, which had previously prohibited ST from providing vaccines to these individuals. By becoming a VFC provider, ST was able to provide essential vaccination services to local adolescents with the fewest resources and lowest access to care, and therefore the greatest need. This finding has significant potential for impact on a larger scale, given that rural populations are more likely to have high poverty and high reliance on Medicaid [27, 28]. Previous work to provide pharmacy HPV vaccinations with financial assistance have demonstrated greater success than those presented above. In a pilot study by Navarrete et al. along the U.S./Mexico border in a university-based pharmacy among the uninsured and underinsured student population, partnering with the Merck vaccine prescription assistance program (MVPAP), enabled pharmacist to administer 167 doses of the HPV vaccine and substantially increase vaccination rates in a medically underserved population over the course of 2 years [45]. The authors attribute their success, in part, to the unique use of MVPAP, which is similar to VFC enrollment in that it makes pharmacy vaccination programs financially viable. Interventions like the one described here could greatly reduce vaccination disparities by providing not only access to care, but specifically meeting the needs of rural, low-income populations.

The potential for increased vaccination among low-income children and adolescents in rural settings through the current strategy is evident in the uptake rates. In total 166 vaccinations were administered to 89 adolescents, over 70% of whom were VFC patients. Provision of other adolescent vaccines was secondary to our primary outcome, but the significant uptake indicates potential for positive impact across an array of vaccines. Additionally, the racial breakdown of participating patients (over 60% Black/African American) offers implications for increasing health equity and possibility to decrease racial disparities in vaccination.

Finally, the indirect results ST attributed to the intervention such as increases in prescriptions, revenue, and community engagement were unanticipated but welcomed outcomes. These added benefits from participation in the intervention offer additional incentives for pharmacies to sustain the program and for other pharmacies to enroll. The success of this intervention demonstrates the potential of these strategies—particularly that of VFC enrollment—on increasing HPV vaccination rates in rural settings while providing mutual benefits to community pharmacy partners.

Due to the present study’s primary focus on feasibility and single study sample, the generalizability of these findings is limited. Next steps will include a randomized-controlled trial to determine generalizability. This future work will also employ a more rigorous evaluation strategy. Although not a limitation noted in the current study, future work will address physician hesitancy to pharmacy-administered vaccines, which may result from concerns regarding inadequate communication and documentation, ability for pharmacists to monitor potential side effects, and discouraging patients from attending provider well visits. In a national survey, 60% of family medicine physicians believed pharmacy-administered HPV vaccines created a good solution to create more vaccination opportunities while only 50% of pediatricians did [46]. In future studies, it will be necessary to include rural community family practitioners and pediatricians in collaborative efforts to reduce these provider concerns while maximizing opportunities to increase HPV vaccination.

## Conclusions

The current work presents opportunities for interdisciplinary collaboration between pharmacies and providers which could prove extremely beneficial in rural, medically underserved areas. Next steps will expand this intervention to determine generalizability and broader dissemination capability. Ultimately, this intervention offers significant promise for increasing HPV vaccination in rural areas and should be considered as a leading strategy for dissemination for those seeking to reduce rural and urban vaccination disparities.

## Data Availability

The datasets used and/or analyzed during the current study are available from the corresponding author on reasonable request.

## Abbreviations

HPV: Human papillomavirus
VFC: Vaccines for Children
USDA: United States Department of Agriculture
ADPH: Alabama Department of Public Health
ImmPRINT: Immunization Division Patient Resources with Integrated Technology
ACIP: Advisory Committee on Immunization Practices
Tdap: Tetanus, diphtheria, and pertussis
ST: Stacie Turberville, owner/pharmacist
MVPAP: Merck vaccine prescription assistance program
